# Dental records: An overview

**DOI:** 10.4103/0974-2948.71050

**Published:** 2010

**Authors:** B K Charangowda

**Affiliations:** *K. V. G. Dental College, Sullai, Dakshina Kannada, Karnataka, India*

**Keywords:** Dental records, forensics, medicolegal

## Abstract

Dental records consist of documents related to the history of present illness, clinical examination, diagnosis, treatment done, and the prognosis. A thorough knowledge of dental records is essential for the practicing dentist, as it not only has a forensic application, but also a legal implication with respect to insurance and consumerism. This article reviews the importance of dental records in forensics.

## Introduction

Forensic odontology is the application of the art and science of dentistry to resolve matters pertaining to the law. Some of the diverse facets of this unique discipline can range from the identification of human remains to mass disaster management, from the assessment of bite marks and patterned skin injuries to the use of dental materials in the examination of evidence.[1]

A dental record is the detailed document of the history of the illness, physical examination, diagnosis, treatment, and management of a patient. Dental professionals are compelled by law to produce and maintain adequate patient records. With the increasing awareness among the general public of legal issues surrounding healthcare, and with the worrying rise in malpractice cases, a thorough knowledge of dental record issues is essential for any practitioner. The ability of clinical practitioners to produce and maintain accurate dental records is essential for good quality patient care as well as it being a legal obligation. The dental record provides for the continuity of care for the patient and is critical in the event of a malpractice insurance claim.[2]

Comprehensive and accurate records are a vital part of dental practice. Good record keeping is fundamental for good clinical practice and is an essential skill for practitioners. The primary purpose of maintaining dental records is to deliver quality patient care and follow-up. Dental records can also be used for forensic purposes and have an important role in teaching and research, as well as in legal matters. The code of practice on dental records documents the minimum requirements for recording and maintaining dental records and describes some of the underlying principles to be applied by the practitioners in their record keeping.

## Patient Record

**Figure 1:**
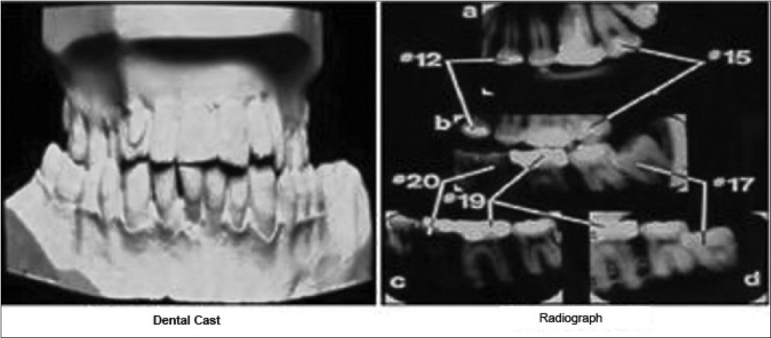
Dental records

**Figure 2:**
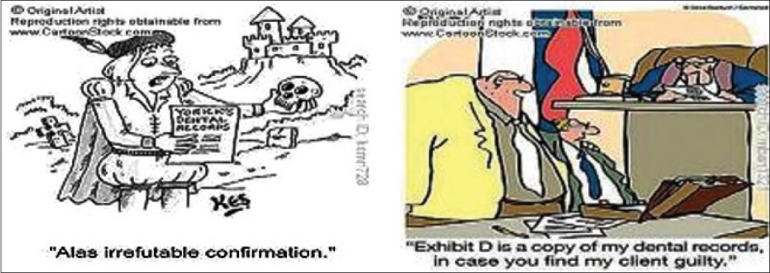
Cartoon on significance of dental records

The record may consist of several different elements, which include written notes, radiographs, study models [Figures 1–2], referral letters, consultants’ reports, clinical photographs, results of special investigations, drug prescriptions, laboratory prescriptions, patient identification information, and a comprehensive medical history. Obviously this is a large amount of information and it is essential that a practitioner maintains this in an easily accessible manner.

The information in the dental record should primarily be clinical in nature. The record includes a patient’s registration form with all the basic personal information. The dental team should be very meticulous and thorough in the dental office record keeping tasks. All information in the dental record should be clearly written, and the person responsible for entering new information should sign and date the entry. The information should not be ambiguous or contain many abbreviations. In practices with more than one dental practitioner, the identity of the practitioner rendering the treatment should be clearly noted in the record. All entries in the patient record should be dated, initialed, and handwritten in ink and / or computer printed. Although no specific color of ink is required, any copy of the record should be easy to read. Handwritten entries should be legible.

Within the written notes the following are examples of what is typically included in the dental record:[3,4]

1. Identification data — name, date of birth, phone numbers, and emergency contact information.
2. Dental history
3. Clinical examination to include an accurate charting
4. Diagnosis
5. Treatment plan
6. Documentation of informed consent
7. Medical history — a thorough investigation, to include a minimum of:-
  - Name and phone number of physician
  - Dentists’ own evaluation of patient’s general health and appearance
  - List of systemic disease — diabetes, rheumatic fever, hepatitis, and the like
  - Any ongoing medical treatment
  - Any bleeding disorders, drug allergies, smoking and alcohol history
  - Any cardiac disorders
  - Relevant family medical history
  - Pregnancy
  - Physical and emotional tolerance for procedures

No financial information should be kept in the dental record. Ledger cards, insurance benefit breakdowns, insurance claims, and payment vouchers are not part of the patient’s clinical record. Financial records should be kept separately from the dental record. Other information best left out of the record would be personal opinions or criticisms. Do document a patient’s refusal to accept the recommended treatment plan and cancelled appointments.

The outside cover of the chart should only display the patient’s name and / or account number. Use of abstract is advantageous in the in-office system (color or symbol coding), so that only your office staff will be able to decipher it. For all offices, a single sticker on the outside cover can alert the team to look on the inside for important information regarding allergies, medications, antibiotic pre-medications, and clinical conditions that can affect dental treatment. All medical notations inside the chart, to be seen only by the authorized personnel.

### Maintenance of dental records

Most dentists make notes on paper dental records. However, many more dentists are making use of computerized filing systems to maintain patient dental records. Electronic records have great quality and patient-safety benefits, and will likely increase as more dental clinics and hospitals become computerized. Many dental clinics use the traditional paper charts; the traditional filing systems are labeled with the following information,

- Patient’s surname
- Patient’s first name
- Patient’s middle name
- Patient’s degree or seniority (i.e., Senior, II)

The files are then arranged in a way for easy retrieval — usually in a lateral, open-shelf filing system.[5]

#### Color Coding

Many dental offices use a color-coded filing system for patient record files. Color-coded labels — usually the first two letters of the patient’s last name and active date of treatment — are placed on the patient’s file. This can help make record retrieval fast and easy.

#### Active and Inactive

Most offices have two categories of patient records files: (1) Active and (2) Inactive.

Active files hold the records of patients currently having their dental care provided by the practice. Inactive patients are considered to be those who have not returned for 24 months. Keep files of active patients on-site. These records should be conveniently located in the office.

Inactive files hold the records of patients who have been treated in the office in the past, but are not currently under care in the office. These files are generally located in the office, but in a remote area.

A system should be established in the office to identify a change from active to inactive status on a timely basis. All records, active and inactive, should be maintained carefully to be certain that they are not destroyed or lost.

Lawney describes a simple ten-step procedure to ensure that your records are adequate. A modified and expanded version, appropriate to the National Health Service and UK dentistry, which has been followed in UK is as follows,[2,3] (adopting the same in our country will be very helpful)

1. Use a consistent style for entries — the appearance of the record is enhanced by using the same colour and type of pen, use the same abbreviations and notations, and so on
2. Date and explain any corrections — it may be a fatal error in a malpractice case if records appear doctored in any way. These unexplained corrections can undermine the credibility of the entire record and of the treating dentist
3. Use single-line crossout — this preserves the integrity of the record and shows that you have nothing to hide
4. Do not use correction fluids — not only is this messy, but it is conspicuous and may indicate that there has been an attempt to hide information.
5. Use ink — pencil can fade and opens up the question of whether or not the records have been altered.
6. Write legibly — an illegible record may be as bad as no record at all. Difficult to read entries can lead to guesswork by others and this may not be favorable to you.
7. Express concerns about patient needs — by doing this you are documenting that you have listened, empathized, understood, and acted upon the wishes of your patient. It also enables an explanation to be given should a patient’s wishes be unobtainable or unrealistic, and can help instantly diffuse a malpractice case. Use quotations to indicate patient comments as distinct from your own.
8. Never write derogatory remarks in the record — Superfluous entries only serve to convey a feeling of unprofessionalism and may create doubts regarding the overall credibility of the remainder of the record. Negative views about patients, such as their failure to follow your advice or attend appointments, should be recorded in a dispassionate and objective manner.
9. Document fully — there is no need to be sparse with notes, a detailed explanation is always better than one lacking information.
10. Only use accepted abbreviations for treatments — this is helpful both in a malpractice situation and also when transferring records to a different dentist for referral, prior approval or a change in dentist.
11. Collate documents — insurance details and other materials from third parties should be separate from those items that pertain directly to patient care.
12. Maintain a chronological order — the use of a hole punch and metal retainer clips on the top of the record may be helpful to keep loose sheets organized.

By following these steps the production of accurate and defensible records is possible.

## Record Management

The recording of accurate patient information is essential to dentistry. The dental record, also referred to as the patient chart, is the official office document that records all diagnostic information, clinical notes, treatment given, and patient-related communications that occur in the dental office, including instructions for home care and consent to treatment. Protecting health information — and diligent and complete record keeping — is extremely important for many reasons,

Care for the patient: Patient records document the course of treatment and may provide data that can be used in evaluating the quality of care that has been provided to the patient.[6]

Means of communication: Records also provide a communication between the treating dentist and any other doctor who will care for that patient. Complete and accurate records provide enough information to allow another provider who has no prior knowledge of the patient to know the patient’s dental experience.

Defense of allegations of malpractice: Besides, the dental record may be used in a court of law to establish the diagnostic information that was obtained and the treatment that was rendered to the patient. This information helps in determining whether the diagnosis and treatment conformed to the standards of care in the community.

Aid in the identification of a dead or missing person: Another way the dental record may be used is to help provide information to appropriate legal authorities that will aid in the identification of a dead or missing person. The most common element of forensic dentistry that a general practitioner is likely to encounter is to supply antemortem (before death) records to a forensic odontologist.[6]

### Retention and storage

There is usually a different requirement for the retention of records of children. These records must be kept for a certain period after the child becomes a major. The dental office should have a records retention policy and all the staff should understand it. The office’s professional liability insurance company will likely have recommendations about retention.

Dental records may be preserved on microfilm or microfiche, stored with a records storage service (fairly common in many jurisdictions) or scanned for electronic storage. The great benefit of storing records electronically or on microfilm or microfiche is that they take up less space than paper records. Diagnostic and / or treatment casts may be photographed and stored in some cases. However, prior to completely converting the records to one of these methods, a dentist should consult with his / her own attorney and a professional liability insurance company.

The accurate health / dental history may provide important and valuable information for the dentist, prior to beginning treatment. All dentists should take health histories initially and update the same periodically as necessary. Dentists have a responsibility to obtain and maintain the current health histories of patients. Team members are most often responsible for having patients complete their health / dental history forms, but that is only part of the process. It is also important that a patient understands the questions, provides appropriate answers, and signs the completed form. A health history form provides a starting point for the dental team to fulfill its professional obligations.[5,7]

The NHS Terms of Service, state that dental records should be kept for a period of two years. The Regulations state that treatment records, radiographs, photographs, and study models should be retained after the completion of any course of treatment and care, under a continuing care or capitation arrangement for this period. There are strict time limits applied to such actions,[3]

1. Within three years of the date when the cause of action occurred
2. Within three years of the patient’s date of knowledge that the treatment may have been negligent
3. If a claim is based upon a Breach of Contract, the action must be raised within five years in Scotland and six years in England and Wales

It is therefore possible that a claim for negligence could happen many years after the event, and that retention of records for the minimum of two years is inadequate. The defense organizations suggest that records be kept permanently. This is often impossible due to space constraints and so the advice given by defense organization is as follows,

1. Treatment Records, X-rays, Study Models, and Correspondence is to be retained for 11 years after the completion of treatment
2. For children, retention of records until the patient is 25 years old
3. Orthodontic Models — retain the original pre- and post-operative models permanently, discard any intermediates after a period of five years.

The storage area of these records should be secure and access strictly controlled. By following these guidelines the dental records of a patient will be available whenever they are needed. Following these guidelines will be very supportive for forensics at our place.

### Confidentiality

Dentists are in a privileged position to learn a lot about their patients and this knowledge is acquired under the assumption that it is confidential. Confidentiality encourages open and honest communication, enhancing the dentist–patient relationship, and encourages respect for patient autonomy and privacy.

There are certain circumstances when information can be disclosed and they include,

1. Sharing of relevant information with other healthcare professionals involved in a patient’s treatment
2. Information may be passed to a third part if the patient or legal adviser gives written consent, for example, an insurance company
3. Where information is requested about a deceased patient and consent of the estate or relative is sought and there is an investigation of sudden, suspicious or unexplained death
4. Information is required in the preparation of legal reports containing only relevant dental treatments
5. Access to dental records by the police. Search and seizure warrants may not include dental records, and therefore should be carefully checked
6. Clinical research protocols and peer review procedures. The name of the patient must be kept confidential. If information is to be used for teaching purposes then the patient’s consent must be obtained

The area of confidentiality of childrens’ dental information can be confusing. Those individuals of 16 years and older should be considered adults, however, for those who are 16 years and under, the dentist still has a duty of care and therefore confidentiality to the child. This duty is combined with a duty to the parents, especially in the area of consent to treatment. Children who are victims of abuse require special management and the dentist may have an overriding responsibility to break confidentiality and report their findings to the appropriate authorities.

Special guidelines exist for patients with AIDS / HIV and sexually transmitted diseases. Strict confidentiality must be maintained when dealing with these individuals. Disclosure of such information could lead to a complaint of serious professional misconduct.[8,9]

## Forensic Uses of Patient Records

Forensic dentistry is the overlap of the dental and legal professions. The most common element of forensic dentistry that a general practitioner is likely to encounter is to supply antemortem (before death) records to aid in personal identification. Forensic dentists are frequently called upon to identify the remains of individuals who cannot be identified visually. This encompasses a large number of situations such as burnt, grossly decomposed or mutilated remains. The identification is normally carried out by the comparison of antemortem (before death) and postmortem (after death) records.[10]

The identification of the deceased individuals is an essential element in the process of death certification and is a crucial component in the investigation of homicides or other suspicious deaths. It is vital to have expeditious and accurate identification both for law enforcing authorities and relatives. Until identification can be confirmed, estates cannot be settled, death benefits cannot be paid, and surviving spouses are unable to remarry. Perhaps of most importance is that the identification of the dead is an essential component of the grieving process and is a necessary part of human dignity in a civilized society.[11]

The police officers in charge of the case will normally call upon the dentist to provide details of dental records. It must be remembered that police officers have no statutory rights to inspect or remove a patient’s records without their consent. However, the law allows for special circumstances and it is reasonable to hand over an individual’s record if it enables them to be identified or excluded. The consent of the nearest relative or estate executor may also be sought if required.[12,13]

The availability of contemporaneous and clear notes is essential in forensic dental identification. If notes are incorrectly dated, it can complicate and even negate a positive identification. It is in such situations where the errors highlighted by Borrman and others can cause crucial mistakes to be made. When a request for records is received the entire record is useful, including such items as laboratory prescriptions and study models. Many documented cases have used the unique pattern of the palatal rugae recorded on an orthodontic study model to identify young individuals with no dental restorations.[9]

The police may require access to an individual’s record for another criminal matter. They may, for example, want to see an appointment book to establish an alibi or time line. In these circumstances a warrant is required if the patient has not agreed to the release, as it can be argued that the release of notes in this instance is not in the patient’s best interest. If in doubt always contact your legal adviser.

## Summary and Conclusion

Doubt about what should be documented or how records need to be kept, practitioners should ask themselves: “Will this action serve the best interests of my patient? Does this action helps in my patient’s safety and the continuity of his or her dental care?” The principles applying to handwritten records also apply to computer records, for example, entries must be date, time, and operator-stamped, all changes made must be traceable, and any codes used must be readily convertible to conventional language. Records must be readily accessible and understandable data needs to be controlled, for example, via use of passwords.

The production, retention, and release of clear and accurate patient records are an essential part of the dentist’s professional responsibility. Success in this task will assist the dentist should a medicolegal claim be made and can assist the police and coroners in the correct identification of individuals.

## References

[1] HTTP://www.wikipedia.org/forensic_dentistry.

[2] Lawney M (1998). For the Record. Understanding Patient Recordkeeping. N Y State Dent J.

[3] http://www.forensicdentistryonline.com.

[4] Collins D (1996). What a dentist should know about oral health record. Nortwest Dent.

[5] http://www.ada.org.

[6] Platt M, Yewe-Dyer M (1995). How Accurate is your Charting?. Dent Update.

[7] Ray AE, Staffa J (1993). The Importance of Maintaining Adequate Dental Records. N Y State Dent J.

[8] Skifkas PM (1996). Guarding the files: Your role in maintaining the confidentiality of patient records. J Am Dent Assoc.

[9] Borrman H, Dahlbom U, Loyola E, Rene N (1995). Quality evaluation of 10 years patient records in forensic odontology. Int J Legal Med.

[10] American Board of Forensic Odontology (1994). Body Identification Guidelines. J Am Dent Assoc.

[11] Nelson GV (1989). Guidelines to the prevention of problems in recordkeeping. Part 1. Pediatr Dent.

[12] Rothwell R, Haglund W, Morton TH (1989). Dental identification in serial homicides: The Green River Murders. J Am Dent Assoc.

[13] Plunkett LR (1997). Managing Patient Records. N Y State Dent J.

